# C-Reactive Protein in Peripheral Blood of Patients with Chronic and Aggressive Periodontitis, Gingivitis, and Gingival Recessions

**DOI:** 10.1155/2015/564858

**Published:** 2015-08-04

**Authors:** Stepan Podzimek, Jaroslav Mysak, Tatjana Janatova, Jana Duskova

**Affiliations:** School of Dental Medicine, First Faculty of Medicine and General University Hospital, Charles University, Karlovo Náměstí 32, Katerinska 32, 121 11 Prague, Czech Republic

## Abstract

CRP is a plasma protein that reflects a measure of the acute phase response to inflammation and is one of the markers of choice in monitoring this response. CRP can be used for the prediction and early detection of periodontal disease. The aim of this study was to compare and evaluate the systemic levels of CRP in the peripheral blood samples of patients with chronic and aggressive periodontitis, gingivitis, and gingival recessions and compare them with periodontal clinical parameters. All patients (*N* = 158) were examined prior to the initiation of periodontal treatment. Patients were divided into four groups. Group A consisted of 26 patients with aggressive periodontitis, Group B consisted of 111 patients with chronic periodontitis, Group C consisted of 13 patients with gingivitis, and Group D consisted of 8 patients with gingival recessions. Our study results indicate that CRP levels increase subsequently with the severity of the periodontal disease and that the bleeding on probing index showed much better positive correlation with the CRP levels compared to the pocket depth index in both periodontitis patients groups, especially in aggressive periodontitis patients.

## 1. Introduction

C-reactive protein (CRP) was discovered in 1930 during a study of patients with* Streptococcus pneumonia* infection [1]. CRP is a plasma protein that reflects a measure of the acute phase response to inflammation and is one of the markers of choice in monitoring this response [2]. CRP participates in the systemic response to inflammation. It is a pattern recognition molecule that binds to specific molecules that are produced during cell death or found on the surfaces of diverse bacterial pathogens. Rapid increase in CRP synthesis within hours after infection suggests its contribution to host defense as part of the innate immune response [3].

CRP is produced in response to many forms of injury other than periodontitis, such as other infections, trauma and hypoxia, and it is regulated by diverse cytokines. CRP levels have an association with smoking, obesity, triglycerides, diabetes, and periodontal disease [4]. Changes in peripheral blood cellular and molecular components can be found in patients with periodontitis due to inflammatory changes of the periodontal tissues [5].

Plasma levels of CRP rise rapidly and markedly as much as 100-fold or more within 72 hours following tissue injury and may increase as much as 1000-fold or more within a longer period after an acute inflammatory stimulus; therefore, CRP is a sensitive marker to evaluate the inflammatory status [3, 6]. Positive correlation between CRP and periodontal disease severity was proved by many studies [7–11], and levels of CRP decrease after nonsurgical periodontal therapy [12–16].

A study by Jayaprakash et al. revealed that the periodontitis group had a higher mean CRP level (2.49 ± 0.47 ng/mL) compared to the gingivitis group (1.40 ± 0.32 ng/mL) and healthy group (0.56 ± 0.20 ng/mL) [10]. The study conducted by Shojaee et al. demonstrated the difference between CRP in healthy subjects and patients with periodontal disease, gingivitis, chronic periodontitis and healthy control. The CRP levels were 5332.62 ± 5051.63 pg/mL in periodontitis patients, 3545.41 ± 3061.38 pg/mL in the gingivitis group, and 3108.51 ± 3574.47 pg/mL in healthy subjects. The statistic analysis showed a significant difference in CRP concentrations between the periodontitis patients and healthy subjects (*p* = 0.045) [17]. The patients with gingivitis and healthy gingiva had lower levels of CRP than the patients with chronic periodontitis. Furthermore, with increasing inflammation, the high-sensitivity CRP levels increased proportionately [18]. It is possible to use CRP in prediction and early detection of periodontal disease [17].

Periodontal disease is an inflammatory disease that affects the soft and hard structures that support the teeth. In its early stage, called gingivitis, the gums become swollen and red due to inflammation, which is the body's natural response to the presence of harmful bacteria. In the more serious form of periodontal disease called periodontitis, the gums pull away from the teeth, and the supporting gum tissues are destroyed. Bone can be lost, and the teeth may loosen or eventually fall out [19].

Most studies have focused on CRP levels in chronic periodontitis, and very few are conducted on patients with aggressive periodontitis [7].

Some types of gingival recessions occur in the absence of periodontal disease. Such gingival recessions are considered mucogingival deformities and are included in the category of developmental or acquired deformities and conditions, according to Armitage's 1999 classification [20]. Gingival recessions can be localized or generalized, and one or more surfaces may be involved [21].

Positive correlation between CRP and periodontal disease severity with particular concern in younger individuals could be a possible underlying pathway in the association between periodontal diseases, and the observed higher risk of cardiovascular disease in periodontitis patients is mentioned in the study by Goyal et al. [7]. The inflammatory mediators, serum elastase, and CRP are all associated with an increased risk of coronary heart disease. Wohlfeil et al. compared these systemic inflammatory mediators in periodontally healthy controls, patients with untreated aggressive and chronic periodontitis. Serum elastase and CRP were significantly elevated in patients with untreated aggressive periodontitis compared to healthy controls as well as systemic inflammatory burden [22]. Patients with aggressive periodontitis have statistically significant elevations in serum CRP levels compared to subjects without periodontitis [23]. Periodontitis is a local inflammatory process mediating the destruction of periodontium triggered by bacterial insult, leading to systemic inflammation in the host. Epidemiologically, it has been modestly associated with cardiovascular diseases with elevated acute phase reactants. The increased serum CRP levels and neutrophils counts in chronic periodontitis subjects suggest an addition to the inflammatory burden of the individual, potentially striking towards an increasing risk of cardiovascular events [24].

Thus, the aim of this study was to compare and evaluate the systemic levels of CRP in the peripheral blood samples of patients with chronic and aggressive periodontitis, gingivitis, and gingival recessions and compare them with periodontal clinical parameters.

## 2. Materials and Methods

### 2.1. Study Population

All patients (*N* = 158) were recruited from the patient pool of the Periodontology Department, School of Dental Medicine, First Faculty of Medicine and General University Hospital, Charles University, Prague, Czech Republic, from 2014 to 2015 and all patients were examined prior to the initiation of periodontal treatment. Inclusion criteria were good general health, no medication, diagnosis of chronic periodontitis or aggressive periodontitis, gingivitis and gingival recession according to the ADA AAP Classification [25], and patient's agreement with CRP level determination from peripheral blood. Exclusion criteria included history of any systemic disease or any other disease manifested locally in oral cavity, current pregnancy or lactation, high blood pressure, sleep disturbances, depression, excessive alcohol use, and smoking recently or in past 10 years. All patients were of Caucasian origin.

Patients were divided into four groups. Group A consisted of 26 patients with aggressive periodontitis, Group B consisted of 111 patients with chronic periodontitis, Group C consisted of 13 patients with gingivitis, and Group D consisted of 8 patients with gingival recessions.

Diagnosis of aggressive and chronic periodontitis, gingivitis, and gingival recessions was based on a detailed clinical examination, medical and dental history, tooth mobility, and radiographic assessment of intraoral X-ray status performed in each patient.

Gingival recession is a noninflammatory periodontal disease; therefore, we used patients with this affection as a control group.

The study was performed with the approval of the Ethics Committees from the First Faculty of Medicine and General University Hospital, Charles University, Prague, Czech Republic. Written informed consent was obtained from all participants in line with the Helsinki declaration before inclusion in the study. This study was performed as a cross-sectional study.

### 2.2. Periodontal Evaluation

Patients with aggressive periodontitis, Group A, had to have at least one tooth with positive bleeding on probing (BOP) and a pocket depth (PD) of >5 mm in all quadrants (excluding the third molars). Patients with chronic periodontitis had to have at least one tooth with positive bleeding on probing (BOP) and pocket depth (PD) of >2 mm in all quadrants (excluding third molars). Pocket depth was assessed by WHO periodontal probe with a cut-off of 11.5 mm from six sites on every tooth present. Bleeding on probing is a sign of inflammation and indicates some sort of destruction and erosion to the lining of the sulcus [26] or the ulceration of sulcular epithelium. The blood comes from the lamina propria after the ulceration of the lining.

In patients with gingivitis, Group C, we used two types of evaluation indices: Papilla Bleeding Index (PBI) and clinical attachment loss (CAL). PBI is a periodontal index that evaluates the gingival status, and bleeding is an indicator of this condition. This index is used for monitoring during treatment of gingivitis [27]. CAL is a sign of destructive (physiologically irreversible) periodontal disease. In gingivitis, inflammation localized to the supracrestal region of the periodontium leads to ulceration of the junctional epithelium. Although this is technically a loss of clinical attachment (because, in health, the epithelium attaches to the surface of the tooth), CAL is used almost exclusively to refer to connective tissue attachment loss. Sites with periodontitis exhibit clinical signs of gingival inflammation and loss of connective tissue attachment. Connective tissue attachment loss refers to the pathological detachment of collagen fibers from the cemental surface with the concomitant apical migration of the junctional or pocket epithelium onto the root surface [28]. The workshop of Australian dental association categorized a general guide for severity on the basis of clinical loss of attachment as follows: slight = 1-2 mm CAL; moderate = 3 to 4 mm CAL; and severe = 5 mm CAL [29]. In our study we measured CAL vestibulary for each tooth with a calibrated Williams probe with a cut-off of 11.5 mm. For the statistical evaluation we used the highest value from the upper and lower jaw.

In patients with gingival recessions, Group D, we used two types of evaluation indices: BOP and CAL. Gingival recessions are noninflammatory affections of periodontium, and this group can serve as control group for Groups A, B, and C, which represent inflammatory diseases of periodontium.

All determined evaluation indices were assessed according to WHO oral health surveys [30].

### 2.3. CRP Determination

CRP levels (mg/L) were measured in capillary blood using device QuikRead go CRP + Hb (Orion Diagnostica Oy, Finland), which works on the principle of photometry and turbidimetry. Capillary blood from the middle finger was collected from the patients before the clinical periodontal examination using a thin glass capillary. The samples were immediately processed and the established values were recorded. All patients were informed in detail about and consented to this marker determination.

### 2.4. Statistical Analysis

For calculations descriptive statistics were used: mean, standard deviation, frequencies, and standard error. This method was used to describe different groups in terms of age, CRP, and other indices present in groups.

For evaluation of the differences between groups, Student's *t*-test was used.

To test linear dependence between the characters, correlation coefficient was calculated and coefficient of determination was used in Figures 2 and 3.

Significance level of 0.05 was used in all tests.

MS Excel 2013 and Data analysis toolpack add-in statistical software were used.

## 3. Results

Characteristics of tested groups are shown in Table 1.

In Group A, mean BOP index was 23.5 ± 27.8% (mean ± SD), and mean PD was 5.7 ± 2.7 mm (mean ± SD). In Group B, mean BOP index was 31.8 ± 30.3% (mean ± SD), and mean PD was 5.2 ± 2.3 mm (mean ± SD). In Group C, mean PBI index was 1.2 ± 1.3 (mean ± SD), and mean CAL was 1.9 ± 1.8 mm (mean ± SD). In Group D, mean BOP index was 0.5 ± 1.1% (mean ± SD), and mean CAL was 4.0 ± 1.5 mm (mean ± SD).

**Figure 1:**
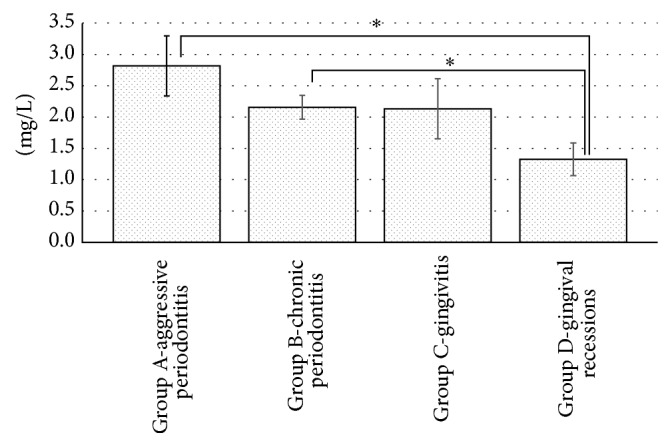
Levels of CRP.

The levels of CRP in Group A patients (2.82 ± 0.48 mg/L, mean ± SE), Group B patients (2.16 ± 0.19, mean ± SE), Group C patients (2.13 ± 0.48, mean ± SE), and Group D patients (1.33 ± 0.26, mean ± SE) are shown in Figure 1. Statistically significant differences were found between Group A and Group D (*p* = 0.01) and between Group B and Group D (*p* = 0.02).

All results are summarized in Table 2, and statistical comparisons of clinical parameters and CRP levels are shown in Table 3.

**Figure 2:**
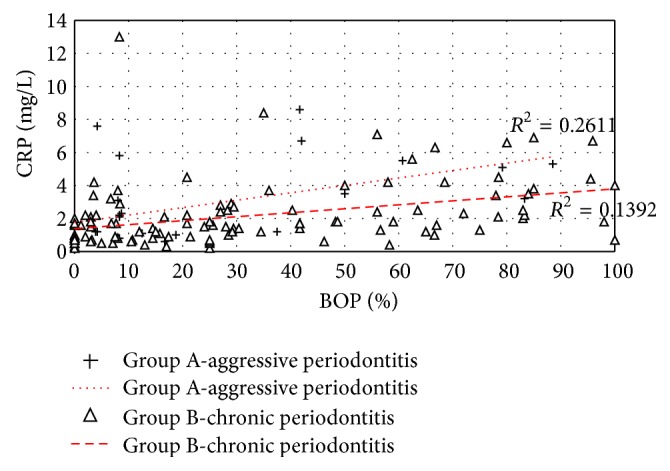
Statistical correlations between BOP and CRP levels in Group A and Group B (*R* ^2^: coefficient of determination).

Statistical correlations between measured BOP indices and determined CRP levels in Group A and Group B are shown in Figure 2. Linear dependence in Group A (coefficient of determination 0.2611) was stronger than in Group B (coefficient of determination 0.1392).

**Figure 3:**
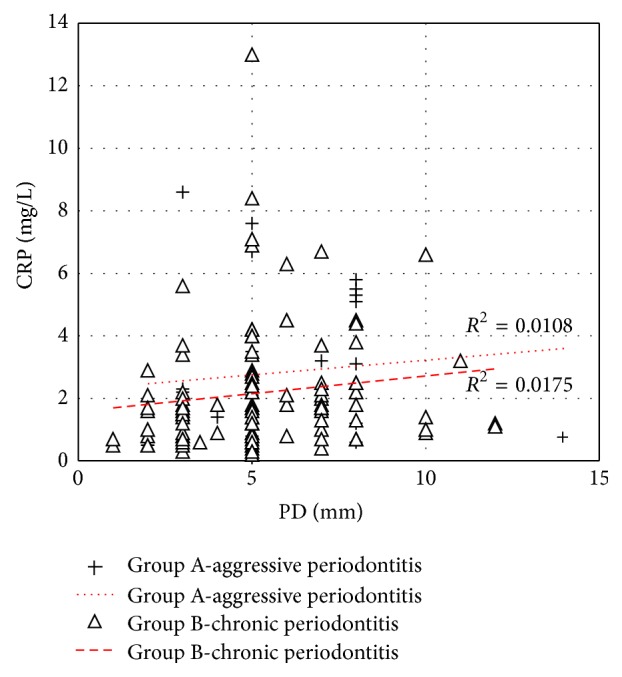
Statistical correlations between PD and CRP levels in Group A and Group B. (*R* ^2^: coefficient of determination).

Statistical correlations between measured PD indexes and determined CRP levels in Group A and Group B are shown in Figure 3. Linear dependence was low in Group A (coefficient of determination 0.0108) as well as in Group B (coefficient of determination 0.0175).

## 4. Discussion

CRP represents a reliable marker of the acute phase response to infectious burdens and/or inflammation [2]. Due to its kinetics, CRP best describes the inflammatory status of human organism [31]. Recent evidence has indicated that patients with severe periodontitis have increased serum levels of CRP compared to unaffected control population [4].

We compared and evaluated the systemic levels of CRP in the peripheral blood samples of patients with chronic and aggressive periodontitis, gingivitis, and gingival recessions. CRP levels may fluctuate with various factors such as high blood pressure, alcohol use, smoking, chronic fatigue, diabetes, sleep disturbances, depression, many other systemic diseases, and pregnancy or lactation [32]. Therefore, we established strong exclusion criteria (determined in Section 2.1) for the patients to be included in this study.

Determined CRP levels were in correlation with the severity of periodontal affection. The highest mean value was found in patients with aggressive periodontitis (2.82 mg/L), and the lowest in patients with gingival recessions (1.33 mg/L). CRP, elastase, lipopolysaccharide binding protein, and IL-6 levels were elevated in patients with untreated aggressive periodontitis compared to healthy control group. Serum elastase and CRP are significantly elevated in patients with untreated aggressive periodontitis. Aggressive periodontitis patients exhibit a stronger systemic inflammatory burden than control patients [22].

Ethnicity has been found to affect the levels of CRP [33] and data in diverse populations are not comparable. A study from the USA recorded CRP levels of 2.05 mg/L in aggressive periodontitis patients with generalized form and CRP levels of 1.1 mg/L in patients with localized form [23]. Another study from the USA showed CRP levels of 4.06 mg/L in subjects with high levels of clinical attachment loss mean [34]. A Swedish study showed median CRP of 2 mg/L in periodontitis patients [35]. In Netherlands, a study reported the highest CRP levels (1.45 mg/L) in patients with generalized form of periodontitis and CRP levels of 1.30 mg/L in patients with localized form [36]. Another study from India showed CRP levels of 7.49 mg/L in aggressive periodontitis patients and CRP levels of 4.88 mg/L in chronic periodontitis patients [37]. Therefore, in our study, only patients of Caucasian origin are included.

Statistical correlations between measured BOP and PD indices and determined CRP levels in periodontitis patient groups, Groups A and B, were established. BOP showed the best positive correlation with the levels of CRP in the aggressive periodontitis group compared to the chronic periodontitis group. In both periodontitis patient groups, BOP showed much better positive correlation with the levels of CRP compared to PD.

Significantly low levels of CRP were observed in the gingival recessions group compared to the aggressive and chronic periodontitis groups. This observation was in accordance with the previous study, where the levels of CRP were significantly lower in gingivitis patients compared to periodontitis patients [38].

A similar phenomenon to what was found in this study regarding CRP levels in patients with aggressive and chronic periodontitis and gingivitis was also published by other researchers. Study of Goyal et al. showed the highest CRP levels in patients with aggressive periodontitis and the lowest values in the group of healthy patients [7]. Other studies showed increased CRP levels in patients with chronic periodontitis compared to patients with gingivitis [10, 17]. Thus, CRP increases with disabilities of periodontium. The comparison of CRP and BOP index is a very important issue. BOP is one of the most important parameters for evaluating the periodontal status of patients with periodontitis. BOP is also the only periodontal parameter which shows the significant relationship with systemic parameters such as CRP and fibrinogen levels, and the white blood cell count, as confirmed in a recent study by Bokhari et al. [39].

Observed association between periodontal conditions and systemic CRP showed that periodontal affections may be one of the factors contributing to systemic inflammation. In the study by Beck et al. it was demonstrated that while attachment loss, pocket depth (PD), and bleeding on probing (BOP) are individually associated with serum soluble intercellular adhesion molecule and CRP, only BOP remains significant for serum soluble intercellular adhesion molecule when all 3 are in the model, and for CRP, only PD remains significant. Both of these clinical parameters were more robust in estimating the degree of systemic inflammation than traditional classifications of mild, moderate, and severe periodontitis or other measures of disease severity such as attachment loss [40].

Novelty of obtained results was based on the comparison of four types of periodontal diseases, as usually two or three types are compared, and on comparison of diverse periodontal indices with subsequently established CRP levels in patient's peripheral blood.

## 5. Conclusion

Our study results show that CRP levels increase subsequently with the severity of the periodontal disease. The lowest CRP levels were found in patients with gingival recessions, increasing in patients with gingivitis and patients with chronic periodontitis, with the highest levels found in aggressive periodontitis patients.

The bleeding on probing index showed much better positive correlation with the CRP levels compared to the pocket depth index in both periodontitis patient groups, especially in aggressive periodontitis patients.

Further studies are needed to clarify this association and the associated confounding factors. In further research, other systemic markers that might be more specific to periodontal disease such as fibrinogen, leptin, white blood cell count, and interleukin-6 should be considered. Changes in their values during the treatment of periodontal disease could lead to improved monitoring of periodontal tissue status during therapy. Elevated levels of such markers of systemic inflammation are connected to both systemic diseases and periodontal diseases. Studying these markers would certainly be beneficial for monitoring periodontal disease therapy.

## Figures and Tables

**Table 1:** Groups of patients: characteristics.

	*N*	Females (F)/males (M)	Mean age ± SD	
Group A-aggressive periodontitis	26	F = 13	36.6 ± 7.6	37.5 ± 7.4
		M = 13	38.4 ± 7.4	

Group B-chronic periodontitis	111	F = 65	57 ± 11.3	55.1 ± 11.4
		M = 46	52.4 ± 11	

Group C-gingivitis	13	F = 10	41 ± 13.1	39.2 ± 12
		M = 3	33 ± 4.4	

Group D-gingival recessions	8	F = 3	53 ± 8.9	44.3 ± 10.3
		M = 5	39 ± 7.4	

**Table 2:** Levels of CRP and clinical parameters in all tested groups.

Group	Number	CRP (mg/l)	BOP (%)	PD (mm)	PBI (%)	CAL (mm)
A	26	2.8 (±2.4)	23.5 (±27.8)	5.7 (±2.7)		
B	111	2.2 (±2.0)	31.8 (±30.2)	5.2 (±2.3)		
C	13	2.1 (±1.7)			1.2 (±1.3)	1.9 (±1.8)
D	8	1.3 (±0.7)	0.5 (±1.1)			4.0 (±1.5)

**Table 3:** Statistical comparisons of clinical parameters and CRP levels in all tested groups (*R* ^2^: coefficient of determination).

	*R* ^2^	Pearson	*p* value
A-PD, CRP	0.0108	0.1039	0.6132
**A-BOP**,** CRP**	**0.2611**	**0.5110**	**0.0076**
B-PD, CRP	0.0175	0.1323	0.1686
**B-BOP**,** CRP**	**0.1392**	**0.3731**	**0.0001**
**C-PBI**,** CRP**	**0.3831**	**0.6190**	**0.0241**
**C-CAL**,** CRP**	**0.3212**	**0.5667**	**0.0434**
D-CAL, CRP	0.0079	0.0889	0.8346
D-BOP, CRP	0.2516	0.5016	0.2053
